# A Longitudinal Trajectory of Loneliness and the COVID-19 Pandemic Among the Oldest-Old Population

**DOI:** 10.1093/geroni/igab046.3649

**Published:** 2021-12-17

**Authors:** Jinmyoung Cho, Shinae Choi, Gelareh Rahimighazikalayeh, Peter Martin, Melinda Heinz, Yeon Ji Ryou

**Affiliations:** 1 Baylor Scott & White Health Research Institute, Temple, Texas, United States; 2 The University of Alabama, Tuscaloosa, Alabama, United States; 3 Baylor Scott & White Health Research Institute, Dallas, Texas, United States; 4 Iowa State University, Ames, Iowa, United States; 5 Upper Iowa University, Fayette, Iowa, United States

## Abstract

Loneliness is significantly associated with health and well-being among oldest-old adults. Due to the outbreak of the COVID-19, physical and social distancing policies might elevate loneliness among the oldest-old population. This study examined the trends and changes in the prevalence of feeling lonely using the 2020 HRS COVID-19 module merged to the 15 waves of the HRS RAND longitudinal datasets from 1992 to 2018. A total of 14,371 respondents, including 614 respondents aged 80 years and older were included. Generalized linear models compared age group differences within the 2020 module. Generalized estimating equations assessed the longitudinal change at the individual level and the trend of feeling loneliness among oldest-old adults from 1992 to 2020. Loneliness was assessed with one item of the CES-D scale (i.e., during the past week, felt lonely). After adjusting for demographic characteristics and health, the results showed that oldest-old adults were more likely to feel lonely compared to younger age groups (18% for 80’s vs. 14% for 50’s) during the early months of the pandemic. A longitudinal trajectory also showed that they feel lonelier than in prior years (19% in 2020 vs. 14% in 2018). However, compared to same-age groups from earlier years, a significantly lower prevalence of feeling lonely was observed (18% in 2020 vs. 27% in 1994). The results show that the outbreak of the COVID-19 may elevate feeling lonely, but the recent cohorts be less lonely than earlier cohorts. Future research should continue to explore protective factors for loneliness among oldest-old adults.

